# Preoperative serum fibrinogen is an independent prognostic factor in operable esophageal cancer

**DOI:** 10.18632/oncotarget.8171

**Published:** 2016-03-18

**Authors:** Shui-Shen Zhang, Yi-Yan Lei, Xiao-Li Cai, Hong Yang, Xin Xia, Kong-Jia Luo, Chun-Hua Su, Jian-Yong Zou, Bo Zeng, Yi Hu, Hong-He Luo

**Affiliations:** ^1^ Department of Thoracic Surgery, The First Affiliated Hospital, Sun Yat-Sen University, Guangzhou, People's Republic of China; ^2^ Guangdong Esophageal Cancer Research Institute, Guangzhou, People's Republic of China; ^3^ Department of Thoracic Oncology, Sun Yat-Sen University Cancer Center, Guangzhou, People's Republic of China; ^4^ State Key Laboratory of Oncology in South China, Sun Yat-Sen University Cancer Center, Guangzhou, People's Republic of China; ^5^ Department of Medical Ultrasonics, First Affiliated Hospital of Jinan University, Guangzhou, People's Republic of China

**Keywords:** Chinese cohort, esophageal cancer, serum fibrinogen, overall survival, disease free survival

## Abstract

In order to fully elucidate the association between serum fibrinogen and prognosis of esophageal cancer, we examined serum fibrinogen concentrations in 1512 patients who underwent esophagectomy by the Clauss method. The impact of fibrinogen on overall survival and disease-free survival was analyzed using the Kaplan-Meier method and Cox proportional hazard models. Hyperfibrinogenemia was significantly associated with older age, male gender, smoking, alcohol consumption, weight loss, advanced pathological T stage and lymph node metastasis. Patients with hyperfibrinogenemia exhibited poor OS (HR=1.20, 95%CI: 1.04-1.38, *P*=0.012) and DFS (HR=1.18, 95%CI: 1.03-1.35, *P*=0.019). Subgroup analysis further exhibited an significant association between hyperfibrinogenemia and poor OS (*P*<0.001), DFS (*P*<0.001) in esophageal squamous cell carcinoma (*P*<0.001) and early pathological stage (I-II) (*P*=0.001). Collectively, this study indicates that preoperative serum fibrinogen is an independent prognostic factor for survival in esophageal cancer.

## INTRODUCTION

Esophageal cancer is one of the most common cancers in the world, with over 480,000 new cases and 400,000 deaths annually, approximately half of whichoccur in China [1]. Despite advances in surgical techniques and the incorporation of new therapeutic approaches, esophageal cancer is still a highly devastating disease with a poor prognosis [2, 3]. Currently, there is no effective biomarkers available for esophageal cancer management, thus, identifying such biomarkers, including prognostic predictors, is in urgent need.

Fibrinogen, a 350-KDa glycoprotein, is synthesized mainly by the liver epithelium [4]. Fibrinogen is recognized as one of several acute phase reactant proteins that are produced during systemic inflammation or tissue injury. After converted to insoluble fibrin by activated thrombin, fibrinogen plays an important role in regulating blood clotting, fibrinolysis, inflammation, wound healing and neoplasia [5]. In cancer, accumulating evidence has demonstrated that serum hyperfibrinogenemia is associated with malignant cell growth, progression and metastasis, such as lung [6], colorectal [7], cervical [8], ovarian [9] and pancreatic cancer [10]. On the other hand, some recent studies indicate that hyperfibrinogenemia is more prevalent in esophageal cancer patients and it contributes to tumor progression, metastasis, poor survival and resistance to chemoradiotherapy [11–13]. However, the significance of serum fibrinogen concentration in operable esophageal cancer patients as a predictor of survival remains understudied. In order to fill this gap, we analyzed a large cohort of Chinese patients to elucidate the potential correlation between serum fibrinogen and prognostic survival.

## RESULTS

### Patient characteristics by fibrinogen

After excluding patients receiving neoadjuvant or adjuvant therapy or with unknown fibrinogen, 1512 consecutive patients with esophageal cancer were included in the study. The median of serum fibrinogen concentration in all patients was 3.45 g/L (range: 0.30–8.00 g/L). Of the patients,1067 (70.6%) had a normal serum fibrinogen concentration (<4.0 g/L), and 445 (29.4%) had hyperfibrinogenemia (≥4.0 g/L). The baseline characteristics of these patients are summarized in Table 1. Fibrinogen levels were significantly associated with age (*P*<0.001), gender (*P*=0.002), smoking status (*P*=0.001), alcohol consumption (*P*<0.001) and weight loss (*P*<0.001). Patients with hyperfibrinogenemia were more likely to be diagnosed with an advanced pathological T stage (*P*<0.001) and N stage (*P*<0.001), to have more metastatic lymph nodes (*P*<0.001) and to have a higher lymph node ratio (*P*<0.001) than those with normal levels. However, there was no significant association between fibrinogen level and histopathology, surgical procedures, differentiation and tumor location (*P*>0.05). We examined serum fibrinogen concentrations according to patient characteristics and found similar results (Table 1).

**Table 1:** Serum fibrinogen level and clinicopathologic characteristics in 1512 patients with esophageal cancer

Characteristic	Patients (%)	Fibrinogen		*P*	Fibrinogen	*P*
	Overall (n=1512)	Normal (<4.0 g/L)	High (≥4.0 g/L)		Median(mean,5^th^-95^th^)	
**Hp**				0.329		0.403
**ESCC**	1305 (86.3)	926 (71.0)	379 (29.0)		3.46(3.57, 2.25-5.41)	
**EA**	164(10.9)	115(70.1)	49(29.9)		3.40(3.57, 2.18-5.53)	
**Others**	43(2.8)	26 (60.5)	17(39.5)		3.67(3.89, 2.10-7.16)	
**Age**				**<0.001**		**<0.001**
**≤58 years**	782(51.7	590 (75.4)	192 (24.6)		3.38(3.47, 2.22-5.31)	
**>58 years**	730 (48.3)	477(65.3)	253 (34.6)		3.55(3.71, 2.27-5.59)	
**Gender**				**0.002**		**0.001**
**Females**	368(24.3)	283 (76.9)	85 (23.1)		3.30(3.41, 2.25-4.91)	
**Males**	1144 (75.7)	784(68.5)	360(31.5)		3.51(3.64, 2.22-5.60)	
**Smoking**				**0.001**		**<0.001**
**Never**	551(36.4)	417(75.7)	134(24.3)		3.29(3.43, 2.15-5.11)	
**Ever (former + current)**	961 (63.6)	650(67.6)	311(32.4)		3.53(3.67, 2.27-5.58)	
**Alcohol**				**<0.001**		**<0.001**
**Never**	1062 (69.9)	793(70.2)	269(29.8)		3.36(3. 47, 2.19-5.25)	
**Ever (former + current)**	450(30.1)	274(60.9)	176(39.1)		3.71(3.84, 2.36-5.81)	
**Weight loss**				**<0.001**		**<0.001**
**No**	830 (54.9)	618 (74.5)	212(25.5)		3.32 (3.48, 2.18-5.40)	
**Yes**	682 (45.1)	449 (65.8)	233 (34.2)		3.60(3.71, 2.32-5.43)	
**Differentiation**				0.974		0.909
**G1**	1015(67.1)	716(70.5)	299(29.5)		3.46(3.59, 2.22-5.44)	
**G2-3**	497(32.9)	351(70.6)	146(29.4)		3.44(3.58, 2.26-5.32)	
**Tumor location**				0.132		0.089
**Upper**	290 (19.2)	208(71.7)	82(28.3)		3.45(3.57, 2.30-5.49)	
**Middle**	769(50.9)	557(72.4)	212(27.6)		3.39(3.52, 2.19-5.34)	
**Lower**	297(19.6)	193(65.0)	104(35.0)		3.61(3.74, 2.27-5.61)	
**EGJ**	156(10.3)	109(69.9)	47 (20.1)		3.43(3.60, 2.21-5.62)	
**pT category**				**0.003**		**<0.001**
**T1-2**	462(30.6)	350(75.8)	112(24.2)		3.20(3.42, 2.10-5.36)	
**T3-4**	1050(69.4)	717(68.3)	333(31.7)		3.55(3.66, 2.29-5.50)	
**pN category**				**<0.001**		**<0.001**
**N0**	775(51.3)	583(75.2)	192(24.8)		3.35(3.47, 2.20-5.17)	
**N1-3**	737(48.7)	484(65.7)	253(34.3)		3.55(3.70, 2.28-5.67)	
**Surgical procedures**				0.222		0.116
**Cervicothoracoabdo-minal**	286(18.9)	202(70.6)	84(29.4)		3.47(3.58, 2.33-5.28)	
**Ivor-Lewis**	206(13.6)	135(65.5)	71(34.5)		3.43(3.56, 2.22-5.41)	
**Left transthoracic**	1020(67.5)	730(71.6)	290(29.4)		3.55(3.72, 2.32-5.85)	
**No. metastatic lymph node( median, IQR)**	0(0-2)	0(0-2)	1(0-3)	**<0.001**	**-**	**-**
**Lymph node ratio, (median, IQR)**	0.00(0.00-0.18)	0.00(0.00-0.15)	0.06(0.00-0.25)	**<0.001**	**-**	**-**

Abbreviations: Hp, histopathology; ESCC, esophageal squamous cell carcinoma; EA, esophageal adenocarcinoma; EGJ, esophagogastric junction; G, grade; IQR, interquartile range

Bold values are statistically significant (*P* < 0.05)

### Univariate and multivariate analysis

**Figure 1:**
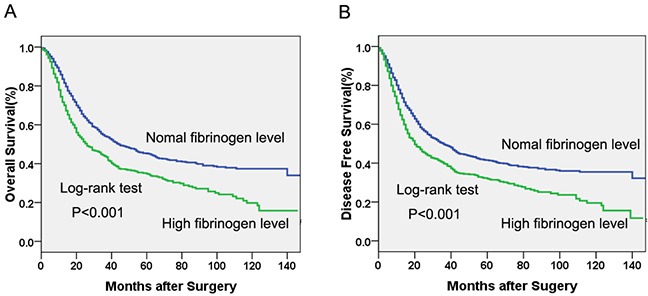
Kaplan-Meier curves of A Overall survival (OS) subdivided by serum fibrinogen level in patients with esophageal cancer, **B.** Disease free survival (DFS) subdivided by serum fibrinogen level in patients with esophageal cancer.

The median times for OS and DFS were 32 months and 26 months, respectively. Univariate survival analysis showed that patients with hyperfibrinogenemia had a significantly poorer OS (53.5 m vs 73.3 m, *P*<0.001, Table 2, Figure 1A) and DFS (50.0 m vs 68.1 m, *P*<0.001, Table 2, Figure 1B) than those with normal levels. As shows in Table 2, patients with older age, male gender, advanced pT caterory, lymph node metastasis, poor histologic differentiation, esophagogastric junction tumor location, weight loss, a history of smoking and alcohol consumption were found to have significantly shorter OS and DFS(*P*<0.05).

**Table 2:** Univariate survival analysis for overall survival and disease free survival in patients with esophageal cancer

Prognostic factor	Overall survival			Disease free survival		
	Mean(m)	HR(95%CI)	*P*	Mean(m)	HR(95%CI)	*P*
**Age**		1.22(1.07-1.40)	**0.003**		1.12(0.99-1.27)	0.079
**≤58 years**	72.8			66.1		
**>58 years**	60.2			57.2		
**Gender**		0.73(0.60-0.83)	**<0.001**		0.75(0.64-0.88)	**<0.001**
**Males**	63.3			59.1		
**Females**	78.0			71.4		
**pT category**		1.85(1.58-2.16)	**<0.001**		1.87(1.60-2.17)	**<0.001**
**T1-2**	86.7			83.0		
**T3-4**	58.1			52.9		
**pN category**		2.70(2.36-3.1)	**<0.001**		2.72(2.38-3.11)	**<0.001**
**N0**	89.3			85.1		
**N1-3**	42.1			37.0		
**Differentiation**		1.53(1.33-1.75)	**<0.001**		1.50(1.32-1.71)	**<0.001**
**G1**	73.5			68.7		
**G2-3**	54.2			49.4		
**Tumor location**		1.10(1.02-1.19)	**0.013**		1.10(1.02-1.18)	**0.012**
**Upper**	64.7			61.1		
**Middle**	71.3			65.7		
**Lower**	61.4			58.0		
**EGJ**	41.2			36.6		
**Weight loss**		1.25(1.10-1.43)	**0.001**		1.22(1.07-1.39)	**0.002**
**No**	72.3			67.2		
**Yes**	61.2			56.8		
**Smoking**		1.31(1.14-1.51)	**<0.001**		1.28(1.11-1.46)	**<0.001**
**Never**	73.8			68.5		
**Ever (former + current)**	62.3			57.9		
**Alcohol**		1.39(1.21-1.60)	**<0.001**		1.36(1.19-1.56)	**<0.001**
**Never**	72.4			67.1		
**Ever (former + current)**	54.8			50.8		
**Fibrinogen level**		1.47(1.28-1.69)	**<0.001**		1.41(1.23-1.61)	**<0.001**
**Normal level**	73.3			68.1		
**Hyperfibrinogenemia**	53.5			50.0		

Abbreviations: EGJ, esophagogastric junction; G, grade; HR, hazard ratio; 95% CI, 95% confidence interval

Bold values are statistically significant (*P* < 0.05).

The Cox proportional hazards regression suggested that serum fibrinogen was an independent prognostic factor in operable esophageal cancer (Table 3). In the final multivariate survival analysis with adjustment for covariates, we found that patients with hyperfibrinogenemia had 20% and 18% higher risks of death (HR=1.20, 95%CI: 1.04-1.38, *P*=0.012) and disease progression (HR=1.18, 95%CI: 1.03-1.35, *P*=0.020) respectively, than patients with normal levels.

**Table 3:** Multivariate survival analysis for overall survival and disease free survival in patients with esophageal cancer

Prognostic factor	Overall survival		Disease free survival	
	HR(95%CI)	*P*	HR(95%CI)	*P*
**Age**	1.25(1.09-1.43)	**0.001**	1.13(0.99-1.28)	0.073
**Gender**	0.85(0.72-1.02)	0.073	0.94(0.76-1.16)	0.561
**pT category**	1.45(1.24-1.79)	**<0.001**	2.38(2.07-2.74)	**<0.001**
**pN category**	2.33(2.01-2.69)	**<0.001**	1.50(1.28-1.75)	**<0.001**
**Differentiation**	1.29(1.12-1.49)	**<0.001**	1.27(1.11-1.45)	**0.001**
**Tumor location**	0.95(0.88-1.03)	0.187	0.96(0.90-1.04)	0.310
**Weight loss**	1.13(0.99-1.30)	0.078	1.08(0.95-1.23)	0.245
**Smoking**	1.03(0.84-1.25)	0.807	1.11(0.96-1.29)	0.177
**Alcohol**	1.21(1.04-1.41)	**0.012**	1.26(1.10-1.45)	**0.001**
**Fibrinogen level**	1.20(1.04-1.38)	**0.012**	1.18(1.03-1.35)	**0.020**

Abbreviations: HR, hazard ratio; 95% CI, 95% confidence interval

Bold values are statistically significant (*P* < 0.05).

### Subgroup analysis

**Figure 2:**
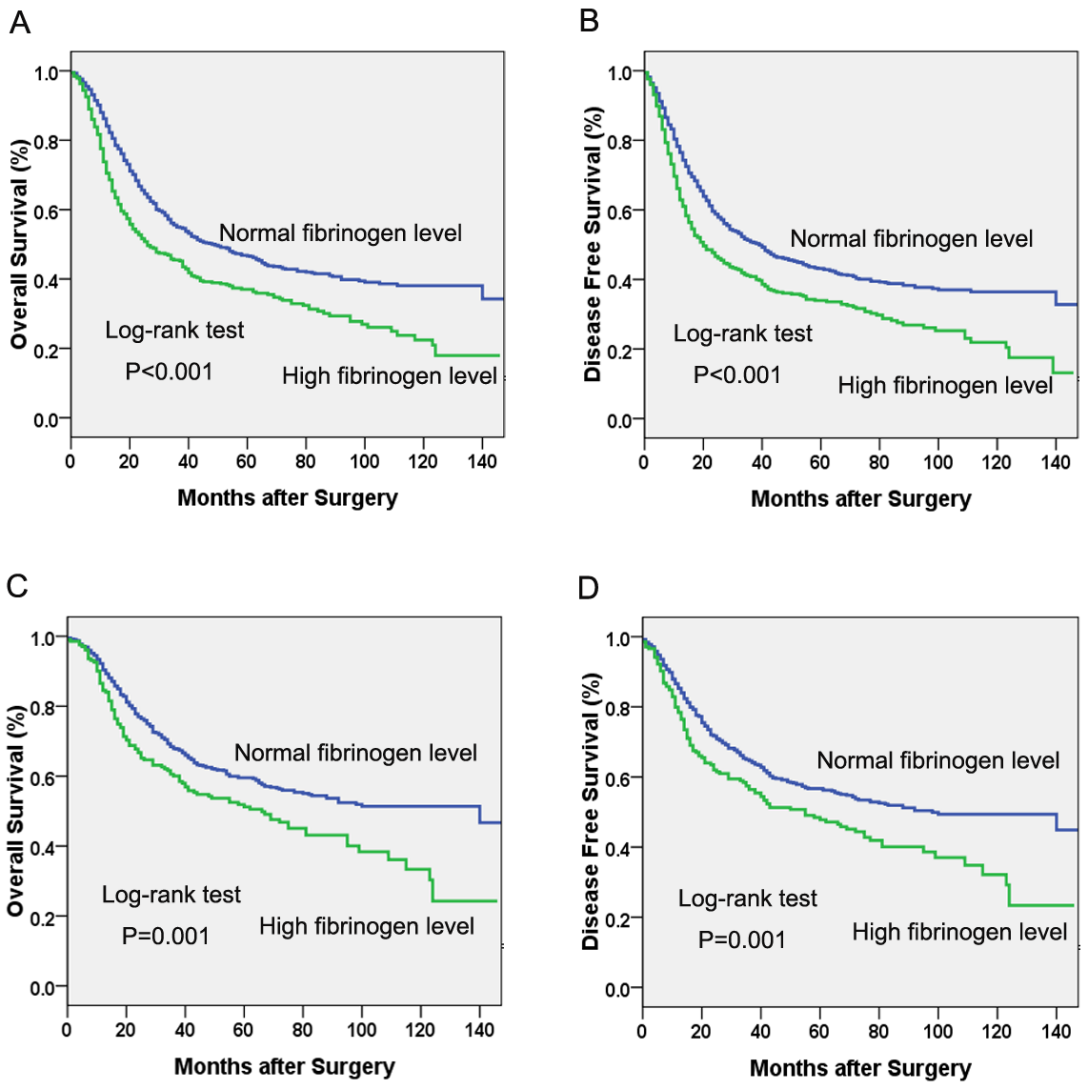
Kaplan-Meier curves of A. Overall survival (OS) subdivided by serum fibrinogen level in patients with esophageal squamous cell carcinoma, **B.** Disease free survival (DFS) subdivided by serum fibrinogen level in patients with esophageal squamous cell carcinoma, **C.** Overall survival (OS) subdivided by serum fibrinogen level in esophageal cancer patients with pathological stage I-II, **D.** Disease free survival (DFS) subdivided by serum fibrinogen level in in esophageal cancer patients with pathological stage I-II.

Univariate survival analyses stratified by histology, age, gender, smoking status, alcohol consumption, weight loss, and pathological stage were performed. We found that hyperfibrinogenemia was associated with decreased OS and DFS in patients with esophageal squamous cell carcinoma (ESCC) (Figure 2A, 2B), young age, older age, male gender, never-smoking, ever-smoking, never alcohol consumption, weight loss history, no weight loss history and early pathological stage (I-II) (Figure 2C, 2D) (*P*<0.05, Table 4). Additionally, the association between hyperfibrinogenemia and decreased OS was also observed in patients with adenocarcinoma(*P*=0.031), female (*P*=0.025) and advanced pathological stage (*P*=0.026). However, there was no significant association between serum fibrinogen and DFS in patients with adenocarcinoma, female or advanced pathological stage (III-IV) (*P*>0.05).

**Table 4:** Subgroup analysis by serum fibrinogen for overall survival and disease free survival in patients with esophageal cancer

Prognostic factor	Overall survival			Disease free survival		
Hp	Mean(m)	Median(m)	*P*	Mean(m)	Median(m)	*P*
**ESCC**			**<0.001**			**<0.001**
Normal level	74.6	48.0		69.7	39.0	
Hyperfibrinogenemia	55.9	26		51.6	20.0	
**EA**			**0.031**			0.106
Normal level	66.5	36.0		58.9	24.0	
Hyperfibrinogenemia	31.7	25.0		29.8	22.0	
**Others**			0.778			0.912
Normal level	45.4	32.0		38.6	18.0	
Hyperfibrinogenemia	47.2	23.0		44.4	15.0	
**Age**						
**≤58 years**			**<0.001**			**0.003**
Normal level	77.1	53.0		69.9	37.0	
Hyperfibrinogenemia	59.7	32.0		55.2	19.0	
**>58 years**			**<0.001**			**<0.001**
Normal level	67.0	41.0		64.1	35.0	
Hyperfibrinogenemia	46.6	24.0		43.5	21.0	
**Gender**						
**Females**			**0.025**			0.055
Normal level	82.0	92.0		74.9	54.0	
Hyperfibrinogenemia	63.7	35.0		60.1	26.0	
**Males**			**<0.001**			**0.001**
Normal level	69.5	40.0		65.0	33.0	
Hyperfibrinogenemia	50.9	25.0		47.3	19.0	
**Smoking**						
**Never**			**0.001**			**0.003**
Normal level	78.8	63.0		72.8	45.0	
Hyperfibrinogenemia	57.4	29.0		54.9	24.0	
**Ever**			**<0.001**			**<0.001**
Normal level	68.2	38.0		63.6	31.0	
Hyperfibrinogenemia	51.4	24.0		47.3	19.0	
**Alcohol**						
**Never**			**<0.001**			**<0.001**
Normal level	78.8	55.0		73.5	43.0	
Hyperfibrinogenemia	53.5	25.0		49.1	20.0	
**Ever**			0.677			0.961
Normal level	56.3	27.0		51.4	22.0	
Hyperfibrinogenemia	52.7	29.0		50.0	21.0	
**Weight loss**						
**No**			**<0.001**			**<0.001**
Normal level	77.2	50.0		72.0	41.0	
Hyperfibrinogenemia	56.6	27.0		51.9	22.0	
**Yes**			**0.001**			**0.003**
Normal level	67.7	36.0		62.4	28.0	
Hyperfibrinogenemia	49.7	24.0		46.7	20.0	
**TNM stage**						
**Stage I-II**			**0.001**			**0.001**
Normal level	92.0	140.0		87.6	98.0	
Hyperfibrinogenemia	73.2	66.0		69.1	55.0	
**Stage III-IV**			**0.026**			0.123
Normal level	42.4	22.0		36.8	17.0	
Hyperfibrinogenemia	36.0	17.0		32.5	14.0	

Abbreviations: Hp, histopathology; ESCC, esophageal squamous cell carcinoma; EA, esophageal adenocarcinoma;

Bold values are statistically significant (*P* < 0.05)

## DISCUSSION

Serum hyperfibrinogenemia has been demonstrated to be associated with tumor progression and unfavorable prognosis in multiple types of cancer, such as lung [6], colorectal [7], cervical [8], ovarian [9] and pancreatic cancer [10]. Some recent studies indicated that increased serum fibrinogen level was significantly associated with elevated risk of ESCC and poor disease prognosis [11, 12]. However, relatively small sample sizes and limited tumor subtype of these studies largely compromised the power of statistical analysis and quality of information. In current study, we performed analysis on a large cohort containing 1512 patients with detailed follow-up data, which not only minimized the potential bias and offset the heterogeneity, but also allowed us to collect information from previously unattended aspects. For example, this comprehensive dataset enables us to expand the analysis on another important subtype of esophageal cancer, adenocarcinoma. This is the first report elucidating the significance of serum fibrinogen in predicting the prognosis of patients of adenocarcinoma in addition to ESCC.

Our study showed that hyperfibrinogenemia was significantly related to advanced pathological staging and poor prognosis. Multivariate analysis further indicated that serum fibrinogen was an independent prognostic factor in patients with esophageal cancer. We also found that hyperfibrinogenemia was closely associated with older age, male gender, smoking, alcohol consumption, weight loss, advanced pathological T stage and lymph node metastasis. These findings were confirmed by the analysis of serum fibrinogen concentration according to patient characteristics. Moreover, patients with hyperfibrinogenemia had elevated metastatic lymph nodes and enhanced lymph node ratio than those with normal levels. Our results were in agreement with previous studies demonstrating that hyperfibrinogenemia was correlated with the depth of invasion and advanced pathological stages in patients with ESCC [11–13]. Recently, Zhang et al [11] reported that increased serum fibrinogen level was associated with pathological T stage and lymph node metastasis. However, the relationship between serum fibrinogen and smoking was not determined in those researches [11, 12]. In the current study, we are the first to report that hyperfibrinogenemia was associated with alcohol consumption and weight loss which were proven to be independent prognostic factors in esophageal cancer in our previous study [14]. Collectively, these findings suggest that serum fibrinogen levels before treatment could potentially be an effective biomarker for TNM staging predictions as a complement to endosonography and integrated fluorodeoxyglucose positron emission tomography-computed tomography in esophageal cancer.

Patients with hyperfibrinogenemia had a decreased OS and DFS compared with those with normal levels. Moreover, multivariate analysis revealed that serum fibrinogen was an independent prognostic biomarker for progression-free and overall survival. Patients with hyperfibrinogenemia had 1.18 times the risk of disease progression and 1.20 times the risk of death of those with normal fibrinogen level. These results are confirmed by previous studies [11–13]. In the study by Zhang et al [11], patients with hyperfibrinogenemia exhibited a 2.54-fold and 1.72-fold increased relative risk of developing distant metastasis and death compared with patients with normal fibrinogen level. More importantly, our multivariate analysis demonstrated that serum fibrinogen level is an independent prognostic indicator of esophageal cancer. Further subgroup analysis found that hyperfibrinogenemia was associated with decreased OS and DFS in patients with esophageal squamous cell carcinoma (ESCC) and early pathological stage (I-II). However, for patients with adenocarcinoma and advanced pathological stage (III-IV), serum fibrinogen was significantly associated with OS but not with DFS. These findings suggested that preoperative serum fibrinogen might serve as a useful biomarker to predict survival in patients with esophageal cancer, especially in those with early stage ESCC.

On the other hand, this study might suffer from several limitations. First of all, although large number of populations was included, our study was a retrospective study focusing on Asian population, which may lead to selection bias. Second, information on post-treatment recurrence was insufficient, which might result in loss of information. In the future, more comprehensive and prospective clinical studies as well as laboratory researches are needed to determine the biological function and prognostic role of serum fibrinogen in esophageal cancer.

## MATERIALS AND METHODS

### Patients

We identified consecutive patients with esophageal cancer who underwent surgical resection at Sun Yat-sen University Cancer Center between December 2000 and December 2008 [14]. All patients were newly confirmed to have esophageal cancer and had not received treatment. Patients were excluded based on the following criteria: history of other cancer; prior neoadjuvant or adjuvant therapy; concomitant disease suspected of influencing serum fibrinogen concentrations, such as severe hypertension, liver disease or a blood coagulation disorder; and history of aspirin or other acetylsalicylic acid use within 1 month before treatment. Patient characteristics were collected via a retrospective medical record review using a standardized data collection form. The surgical procedure was performed as previously described in our study [14, 15]. Esophagectomy with standard or extended dissection of the thoracic and abdominal lymph nodes was executed in patients with no evidence of metastatic disease, including cervical or celiac lymph node metastases. Pathologic stage was determined according to the 7th edition AJCC staging system [16]. The study was approved by the Ethics Committee of Sun Yat-sen University Cancer Center. All patients provided a written informed consent before surgery.

### Follow-up

All patients received standardized follow-up at 3-month intervals for the first 2 years after surgery, a 6-month interval in the third year and yearly thereafter. Follow-up time was calculated from the date of surgery to the event or the date of the last contact. Follow-up continued until June 2012. The primary endpoint was overall survival (OS), which was calculated from the time of surgery to the time of death from any causes. The second endpoint was disease-free survival (DFS). DFS was calculated from the time from surgery to the first recurrence of index cancer or to all-cause death.

### Serum fibrinogen measurement

Each patient provided 4-mL of pretreatment blood. The serum was separated within 30 min after the blood samples were collected. Fibrinogen was measured by the Clauss method using Diagnostica Stago equipment and reagent according to Diagnostica Stago guidelines (Asnieres, France). According to the instructions, a serum fibrinogen concentration <4.0 g/L was considered normal, and a concentration ≥4.0 g/L was defined as hyperfibrinogenemia.

### Statistical analysis

Statistical analysis was performed using SPSS 16.0 for Windows software system (SPSS Inc, Chicago, IL). The serum fibrinogen concentration was analyzed as a continuous variable and as a categorical variable after grouping by normal and hyperfibrinogenemia. The Mann Whitney U-test and a chi-square test were performed to evaluate the associations between clinicopathological variables and serum fibrinogen levels, respectively. Survival curves were calculated by the Kaplan-Meier method and analyzed by log-rank test. Multivariate analysis was performed using Cox's proportional hazards regression model with a forward stepwise procedure (the entry and removal probabilities were 0.05 and 0.10, respectively). A significant difference was declared if the *P* value from a two-tailed test was less than 0.05.
